# What can challenging reproductive contexts tell us about the rat’s maternal behavior?

**DOI:** 10.3389/fnbeh.2023.1239681

**Published:** 2023-07-14

**Authors:** Daniella Agrati, Natalia Uriarte

**Affiliations:** ^1^Sección Fisiología y Nutrición, Facultad de Ciencias, Universidad de la República, Montevideo, Uruguay; ^2^Laboratorio de Neurociencias, Facultad de Ciencias, Universidad de la República, Montevideo, Uruguay

**Keywords:** postpartum estrus, overlapping litters, maternal motivation and behavior, sexual motivation, maternal flexibility

## Abstract

Maternal behavior in mammals encompasses a complex repertoire of activities that ensure the survival of the offspring and shape their neural and behavioral development. The laboratory rat has been employed as a classic model for investigating maternal behavior, and recently with the use of advanced techniques, the knowledge of its neural basis has been expanded significantly. However, the standard laboratory testing conditions in which rats take care of a single litter impose constraints on the study of maternal flexibility. Interestingly, the reproductive characteristics of this species, including the existence of a fertile postpartum estrus, allow us to study maternal behavior in more complex and ethologically relevant contexts, even in laboratory settings. Here we review how maternal and sexual motivations interact during the postpartum estrus, shaping the behavioral response of females according to the presence of the pups and males. Next, we describe how impregnation during the postpartum estrus creates a new reproductive context in which mothers simultaneously care for two successive litters, adapting their responses to different behavioral and physiological demands of pups. These findings illustrate the behavioral adaptability of maternal rats to pups’ needs and the presence of other reinforcers, as well as its dependence on the context. In our view, future perspectives in the field, by incorporating the use of cutting-edge techniques, should analyze maternal flexibility and its neural substrates in models that incorporate complex and challenging contexts. This approach would allow a more comprehensive understanding of brain circuits involved in the adaptive and flexible nature of parenting.

## Introduction

Maternal care is a highly conserved behavior in evolution and, although it is present in several vertebrate and invertebrate species, it is most developed and is universal in mammals. In this group, the mother and her offspring forge a long-lasting bond immediately after (or in some species before) parturition. By far, this is the most common enduring bond in mammals, and, importantly for the young, it represents the first social bond in their life (Hrdy, 2009; Numan and Young, 2016; Pereira and Ferreira, 2016). Maternal care received during this first social experience will not only grant nutrition and protection, ensuring their survival, but it will also shape the developmental trajectories of the offspring (Caldji et al., 1998; Champagne et al., 2003; Uriarte et al., 2007, 2014; Zuluaga et al., 2014). Therefore, maternal behavior (MB) must be robust and contingent on the demands of the offspring, but also on the physiological state of the mother and the context in which it occurs.

In the rat, postpartum females display a series of direct caretaking activities of the young that are well characterized and include retrieving the pups to the nest if they are displaced, licking and arranging them inside the nest, and eventually adopting a reflexive posture to nurse them. The expression of these behaviors, although stereotyped, varies according to the needs of the offspring (Reisbick et al., 1975; Hansen et al., 1993; Pereira and Ferreira, 2006; Pereira et al., 2008; Pereira and Morrell, 2011; Grieb et al., 2018). Indeed, as the postpartum advances and the pups become more autonomous, the duration and frequency of maternal responses progressively decline (Reisbick et al., 1975; Cramer et al., 1990; Giovenardi et al., 2000; Pereira and Morrell, 2009). Moreover, although pups have a strong incentive value for postpartum rats, mothers might modify their MB to cope with other incentives such as an intruder in the home cage or food (Mayer et al., 1987; Kinsley et al., 2014). This evidence illustrates the adaptability of MB to pup characteristics and context, however, this maternal flexibility may often be masked in the standard laboratory testing conditions in which rats are exposed to pups from a single litter in their home cages. Interestingly, the reproductive characteristics of this species provide us with the opportunity to study MB in more complex and ethologically relevant contexts, even in laboratory settings.

As in most rodent species, a few hours after parturition female rats show a fertile postpartum estrus (PPE), a brief period of time when females are simultaneously maternally and sexually motivated (Connor and Davis, 1980a; Gilbert et al., 1980; Lonstein and Stern, 1998; Agrati, 2022). In natural conditions the successful mating during this period and the delayed dispersal of the young result in the temporal overlapping of successive litters within the maternal nest (Calhoun, 1963; Gilbert et al., 1983). This situation can be successfully replicated in semi-natural conditions and the laboratory (Gilbert et al., 1983; Uriarte et al., 2008), giving us the possibility to study the coexistence of maternal and sexual motivations during the PPE, as well as the adaptation of MB of mothers simultaneously caring for two overlapping litters with different physiological demands and behavioral capabilities.

## Mothering during the postpartum estrus

Pups are the “apple of the eyes” of maternal rats. Postpartum females prefer to spend time in an environment associated with mother-pups interaction (Fleming et al., 1994; Wansaw et al., 2008), and insatiable lever-press if this action results in the delivery of pups (Wilsoncroft, 1968; Lee et al., 1999). Maternal motivation appears invulnerable, particularly in the first postpartum week, when lactating rats develop conditioned place preference to a compartment associated with pups, but not to a compartment associated with food (Fleming et al., 1994), and even prefer a pup-associated chamber to a cocaine-associated chamber (Mattson et al., 2001; Pereira and Morrell, 2010). Can another stimulus compete with the pups for the attention of the maternal rat? The PPE, when females are simultaneously maternally and sexually motivated (Gilbert et al., 1980; Agrati, 2022), represents an excellent period for exploring how maternal motivation interacts with sexual motivation.

Postpartum estrus is a common physiological phenomenon since all, or almost all, female rats present it [(Blandau and Soderwall, 1941; Connor and Davis, 1980a,b), observations from our lab]. Females are usually sexually active between 6 and 18 h after delivery, although the maximal expression of sexual behavior occurs around 12 h postpartum (Connor and Davis, 1980a; Carrillo-Martínez et al., 2011).^1^ Gilbert et al. (1980) observed that when given the opportunity to freely interact with both reinforcing stimuli -pups and a male- in a large semi-natural environment, PPE rats exhibit both maternal care and mating, but in separated locations and time windows. Females stay with their pups in the nest area and take care of them until approximately 10 h after parturition, when they leave the pups and copulate with the male away from the nest (Gilbert et al., 1980). The strength of male incentive value for PPE females during these mating bouts is reflected in the little time that females spend with their litters within an ejaculation series, even if the nest area is disturbed (Gilbert et al., 1984).

Given the relevance of the context for females’ sexual responses (Erskine, 1989; Paredes and Alonso, 1997; Guarraci and Frohardt, 2019; Chu and Ågmo, 2023), we wonder how a PPE rat would respond if confronted with the pups and a male in her home cage. As maternal rats vigorously attack intruders in their home cages (Ferreira and Hansen, 1986; Erskine, 1989; De Almeida et al., 2014), while estrous females actively sexually solicit it (Beach, 1976; Pfaus et al., 1999; Blaustein and Erskine, 2002), it was surprising that introducing a male into the home cage of a PPE rat with her pups promoted the merged expression of aggressive responses and sexual solicitation by the female. Therefore, in the context of the home cage, as opposed to the semi-natural environment, the male is perceived as an ambivalent stimulus by the PPE female, who consequently co-expresses maternal aggression and sexual behavior (Agrati et al., 2011). This aggressive response is modulated by the pups. Thus, removing the pups and reintroducing them opposite to the nest, together with the male, increases MB, including pup’s retrieval. This stimulation of MB is associated with an increase of maternal aggression, without affecting sexual behavior (Agrati et al., 2011). In summary, during PPE, when pups and a male are simultaneously available, females are able to flexibly adapt their behavior -maternal behavior and aggression and sexual behavior- depending on the context, even regulating their response according to pup needs.

Unlike what was observed in a semi-natural environment and in the female’s home cage, in a preference test between pups and a male, where no contact with the stimuli was possible, PPE rats ignored the male and preferred the pups (Agrati et al., 2008, 2016). Noteworthy, the time spent near the pups and the attempts made to reach them, were modulated by the female’s maternal motivation. If the maternal motivation of primiparous PPE rats was reduced (by limiting the time of mother-litter interaction), they preferred the male, as opposed to the clear preference for the pups of undisturbed PPE females (Agrati et al., 2016). Moreover, postpartum mothers preferred the pups, while maternal sensitized females^2^ tested in the proestrus chose the male, and multiparous PPE females exhibited a stronger preference for the pups than primiparous rats (Agrati et al., 2008). Thus, factors known to modulate maternal motivation, such as the endocrine profile and previous reproductive experience of the mothers (Fleming et al., 1994; Scanlan et al., 2006; Afonso et al., 2009; Bridges, 2016), modify the behavior of PPE rats in pups vs. male preference test. Together, these studies indicate that the incentive value of pups and males for PPE females influence one another and change depending upon the context. This dynamic value of pups for PPE rats probably sets the basis that underlies the most appropriate behavioral response according to the circumstances.

The mesocorticolimbic dopaminergic system is considered a general motivational system that promotes the activation of motivated behaviors (Salamone and Correa, 2002; Treadway and Salamone, 2022) including maternal and sexual behaviors [for maternal:(Keer and Stern, 1999; Miller and Lonstein, 2005; Numan et al., 2005a; Pereira et al., 2011) and for sexual: (Pfaus, 2009; Coria-Avila et al., 2014) cf. (Paredes and Ågmo, 2004)]. Interestingly, this system has been implicated in effort-related decision-making between two reinforcers (Salamone and Correa, 2002; Hosking et al., 2015; Correa et al., 2016; Treadway and Salamone, 2022). In our preference task between these two social reinforcers, antagonizing dopaminergic neurotransmission diminished the preference for the pups over the male, as well as the attempts that PPE rats made to gain access to them. Remarkably, the same dopaminergic manipulation did not affect the preference of PPE rats for the pups over a female (social stimulus) or the preference for the male over a female (Ferreño et al., 2018b). This result points to a crucial role for dopaminergic activity in shaping the optimal behavioral output when these two reinforcers for PPE rats, the pups and a male, are inaccessible and compete with each other.

The differential effect of this dopaminergic manipulation on PPE females according to the social stimulus competing with pups (purely social or sexual), exemplifies how challenging situations can help elucidate the contribution of a neurotransmitter system, or even a particular brain area, to the behavioral profile of maternal rats. Similarly, we determined that the pups vs. male preference test was sensitive enough to detect motivational differences between PPE rats differing in their internal state, which did not emerge in the maternal behavior home cage test (Agrati et al., 2016). These studies emphasize the importance of confronting motivations to understand how flexible the behavioral response of mother rats can be, and highlight PPE as an excellent model for this purpose. Beyond its relevance for understanding the unique adaptations of the maternal brain, this model may also be useful for exploring the neural basis of the interaction between motivations.

## The challenge of raising overlapping litters

After the birth of a new litter conceived in the PPE, mother rats will simultaneously raise two overlapping litters (OL) with pups with different physiological demands and behavioral capabilities (i.e., nutritional needs, thermoregulation, waste excretion capacities, mobility, and responses to environmental stimuli), a unique reproductive situation first studied by Gilbert et al. (1983) and Stern and Rogers (1988).

Based on undisturbed recording conducted multiple times a day for several days, we reported that mothers direct most of their maternal responses to the newborn pups from the second litter. However, they also continue to provide care for the juvenile pups from the first litter (Uriarte et al., 2008). The mothers do not wean the first litter upon the birth of the new litter, and they invest more in the care of the juveniles compared to same-aged pups raised in single litters (Uriarte et al., 2008). Notably, the presence of the juveniles also influences the OL dam’s MB toward the newborns. They exhibit reduced licking of the newborns and spend more time outside the nest, as compared to mothers rearing single litters (Uriarte et al., 2008). Thus, within the context of OL, mothers display a distinctive behavioral profile, adapting their caregiving activities to the specific characteristics of pups from both litters.

The reduced maternal response exhibited by mothers with OL toward juvenile pups may be attributed to the decreased incentive value of pups at weaning age. This decline in incentive value has been linked to the evolving behavioral capacities and physiological needs of the growing pups. Thus, younger pups tend to be more rewarding for mother rats in specific behavioral tests assessing motivation, such as the conditioned place preference test, compared to older pups (Wansaw et al., 2008). In accordance, we have shown that multiparous mothers during the early postpartum period strongly prefer newborns over juveniles in a preference test without physical access to them, while late postpartum mothers do not express a preference toward either of them (Ferreño et al., 2018a).

Interestingly, OL mothers performed more attempts to gain access to newborns, similar to those observed in early postpartum mothers, suggesting that newborns have a higher incentive value than juveniles for them (Ferreño et al., 2018a). However, OL mothers, similar to late postpartum rats, do not differ in the time spent in the newborns’ and juveniles’ chambers of the maze (Ferreño et al., 2018a). This evidence suggests that juvenile pups have an incentive value for OL mothers, who chose to spend time near them. Therefore, the differential maternal response of these mothers according to the pups’ age observed may be an adaptation to the reduced demands of these more autonomous pups.

The adaptation of the maternal response to the changing needs of growing pups of a single litter throughout lactation has been proposed to rely on changes in the functional role of areas involved in MB regulation, such as the medial preoptic area (mPOA) and the medial prefrontal cortex (mPFC) (Pereira and Morrell, 2009, 2020; Pereira et al., 2011). Transient inactivation of mPOA in early postpartum abolishes the active components of MB such as retrieval, licking and nest building, while this procedure on postpartum days 13–14 restores these behaviors to early postpartum levels (Pereira and Morrell, 2009), indicating a different function of this area throughout the postpartum period. Regarding the distinct subregions of the mPFC, Pereira and Morrell (2011) proposed that the infralimbic subregion (IL-mPFC) has a central role during early postpartum, which wanes as it progresses, whereas the prelimbic subregion (PrL-mPFC) appears to be recruited later in the postpartum.

Similarly, we described specific and distinctive neural activation patterns in OL mothers exposed to newborns or juveniles. Although during the behavioral test the mothers displayed high levels of MB toward the newborns and had minimal interaction with the juveniles, c-Fos expression in the mPOA and the nucleus accumbens was similar after interacting with each type of pup. In contrast, a differential activation according to pups age was quantified in the ventral region of the bed nucleus of the stria terminalis, and in neural areas involved in the flexibility and cognitive processes of the behavior, including subregions of the amygdala and the mPFC (Pose et al., 2019). In line with Pereira and Morrell’s results, the IL-mPFC of OL mothers exhibited the highest c-Fos expression after interacting with newborns. The PrL-mPFC showed high c-Fos expression after interacting with newborns, but even more after interacting with juveniles, pointing toward a particular profile of activation of this area in OL dams with elements that resemble both early and late postpartum periods. This might be part of the unique neural adaptations that allow them to respond adequately to newborn and juvenile pups simultaneously (Pose et al., 2019). Therefore, we posit that the specific profile of activation of the neural circuitry controlling MB enables dams to respond adequately to the requirements of pups with different characteristics and physiological demands. An interesting perspective to deepen this hypothesis includes the analysis of maternal motivation and behavior following the transient inactivation of these specific areas of the maternal neural circuit in mothers taking care of successive litters. Furthermore, delving into the functioning of maternal circuits during OL could help identify differences between areas crucial for being a mother and those that process and evaluate the offspring.

Which factors contribute to this maternal adaptation? Recent studies indicate that the levels of MB toward newborns are lower during late- compared to early postpartum period (Grieb et al., 2018), suggesting a role of the gestation hormonal profile in high maternal response toward neonates. In addition, extensive research conducted throughout the peripartum period demonstrates that gestational and parturition hormones, along with reproductive experience, induce structural and functional remodeling of brain areas associated with motherhood and its adaptations (Kinsley and Bridges, 1988; Leuner and Gould, 2010; Bridges, 2016; Uriarte et al., 2020; Pawluski et al., 2022). To elucidate whether the behavioral adaptation of OL mothers that allows them to care for both types of pups providing differential maternal responses, depends on the endocrine profile of their second gestation and/or on their recent maternal experience, it would be interesting to compare the relative incentive value of newborn and juvenile pups for OL mothers differing in their hormonal exposure and maternal experience.

Studies presented in this section show that the model of rats raising OL provides useful insights into how maternal behavior implicates flexible and contingent processes and can help us better understand the neuroendocrine mechanisms underlying parenting. Additionally, the OL model provides a socially enriching environment for juveniles, promoting increased interaction with their mother and fostering parental behavior toward newborns. As these alterations in early experience can lead to long-term changes in affective behavior (Uriarte et al., 2009, 2014), this model also offers a valuable approach to study the impact of early social environments on individual development and highlights the notion that diverse early social backgrounds can give rise to multiple developmental trajectories.

## Discussion

**FIGURE 1:**
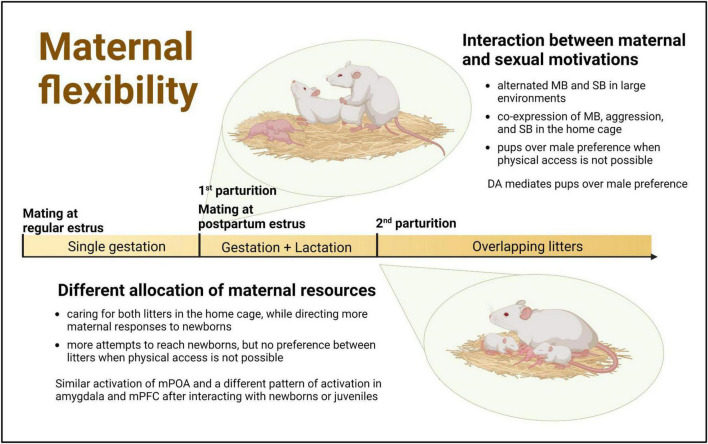
Challenging reproductive contexts in the female rat as a window of opportunity to study maternal flexibility. Within the first 24 h after parturition female rats usually exhibit a fertile postpartum estrus, when they are simultaneously maternally and sexually motivated The successful mating during this period and the delayed dispersal of pups from the first litter can result in the temporal overlapping within the maternal nest of successive litters with different behavioral and physiological demands. MB, maternal behavior; SB, sexual behavior; DA, dopamine; mPOA, medial preoptic area; mPFC, medial prefrontal cortex. Created with BioRender.com.

The findings presented in this article, derived from studies conducted in highly demanding reproductive contexts, show that female rats are able to adapt their behavior specifically to the needs of the pups, the presence of other reinforcers, and the context. This behavioral flexibility allows them to care for and protect their offspring while also engaging in mating or differentially attending to pups of different ages. Consequently, each reproductive context has its own characteristics that not only shed light on, but also facilitate, the examination of complementary aspects of motherhood: coping simultaneously with competing motivations or with different and specific demands of pups (Figure 1).

Traditionally, rodents have been used as models in the study of the neural mechanisms underlying evolutionarily conserved behaviors, including MB. Using these experimental models has proven advantageous as they enable researchers to reduce the number of variables involved, facilitating the study of the neurobiology of this behavior (Ferreira et al., 1987; Fleming and Korsmit, 1996; Numan et al., 2005b; Numan, 2007; Fleming et al., 2008; Pereira and Morrell, 2009, 2011; Olazábal et al., 2013). Based upon these foundations, over the past few decades, technological advances have allowed the identification of neuronal phenotypes and the specificity of their connections, as well as the detection of epigenetic marks in the maternal neural circuitry (Champagne et al., 2006; Fang et al., 2018; Kohl et al., 2018; Mayer et al., 2019). However, as our understanding of this neural circuit continues to evolve, it becomes crucial to test it in situations that closely mimic the complex and natural environmental conditions in which MB actually unfolds, as exemplified by the scenarios mentioned earlier. For example, in comparison to mothers caring for pups in standard conditions, the behavioral flexibility of PPE females when confronted with pups and a male and that of mothers raising OL, likely demands a greater involvement of cognitive functions (Pose et al., 2019), processes depending on the integrity of mPFC circuits and their subcortical connections (Dalley et al., 2004).

Moreover, the refinement in the analysis of classically recorded variables, as well as the incorporation of new ones, is proving a useful insight into the characterization of MB (Carcea et al., 2021; Rivas et al., 2021; Tuncali et al., 2023; Winters et al., 2023), and may be relevant for a more accurate description of the maternal repertoire displayed in challenging contexts. For example, Tuncali et al. (2023), through the analysis of ultrasonic vocalizations of the mother-litter dyad, propose a role for the expression of maternal positive affect in the mother-pup interaction. Will females in PPE similarly vocalize toward a male and their pups? Will this affective communication differ in mother rats during an interaction with younger or older litters?

We believe that future perspectives in this field should integrate advanced techniques such as neuroimaging, optogenetics, and molecular biology and refinement in behavioral analysis with models that incorporate complex and challenging contexts. This integrated approach would provide a more comprehensive understanding of the neural circuits implicated in the adaptive and flexible nature of parenting.

## Data availability statement

The original contributions presented in the study are included in the article/supplementary material, further inquiries can be directed to the corresponding authors.

## Author contributions

DA and NU worked equally on the conception, development of the ideas, and writing of the manuscript. Both authors contributed to the article and approved the submitted version.

## Footnotes

1 A detailed description of female behavior during this period is provided in Agrati (2022).

2 Rats rendered maternal through the continuous exposure to pups, without experiencing gestation and parturition (Fleming and Rosenblatt, 1974).
